# Effectiveness of Therapeutic Patient Education Interventions for Older Adults with Cancer: A Systematic Review

**DOI:** 10.1007/s12603-020-1395-3

**Published:** 2020-05-20

**Authors:** Marie Champarnaud, H. Villars, P. Girard, D. Brechemier, L. Balardy, F. Nourhashemi

**Affiliations:** 1 Geriatric Department, University Hospital, Toulouse, France; 2 UMR 1027 Department of Epidemiology and Public Heatlh- Aging, University of Toulouse III, Toulouse, France; 3 c-Inserm U 558, University Toulouse III, F-31073, Toulouse, France; 4 Centre Hospitalier Universitaire de Toulouse, Place Baylac, URM Post-Emergency Unit, Hôpital de Purpan, 31059, Toulouse Cedex, France

**Keywords:** Geriatric oncology, patient education, counselling, older patient with cancer, literature review

## Abstract

The incidence of cancer increases with age and demographics shows that the population of western countries is dramatically ageing. The new discipline of Geriatric Oncology is emerging aiming at providing tailored and patient-centred support to older adults with cancer. With the development of oral cancer therapy and outpatient treatments, Therapeutic Patient Education (TPE), aiming at enabling the patient and their relatives to cope with the disease in partnership with health professionals, appears to be an interesting and useful tool. The purpose of this paper is to search for evidence of the effectiveness of educational interventions for patients in older adults with cancer. The first screening found 2,617 articles, of which 150 were eligible for review. Among them, fourteen finally met the inclusion criteria: experimental and quasi-experimental studies enrolling older adults (over 65 years old), suffering from cancer and receiving an educational intervention. The types of educational intervention were diverse in these studies (support by phone and web base material). The results appear to be positive on anxiety, depression and psychological distress, patient knowledge and pain. However, data currently available on the effectiveness of a TPE program in Geriatric Oncology is lacking. Further studies are needed to assess the effectiveness of TPE programs adapted to the specific circumstances of the older adult.

## Introduction

### Context

The prevalence of cancer in people over 65 years of age is high and increasing worldwide (1). Cancer is a public health issue in Western countries; by 2020 fifteen million new cases of cancer could appear per year, according to the World Cancer Report, compared to fourteen million in 2012 (1). The prevalence of cancer increases with age and the population is ageing. Theese two facts will cause the total number of older adults with cancer to increase dramatically in the future. For example, in France in 2015, 60,9 % of cancers diagnosed occurred in people over 65 years old and 10,9% occurred in people over 85 years of age (2, 3).

In addition, there are many age-related specificities in cancer management (comorbidities, treatment goals, drug toxicity, adherence to drugs, role of the relative…) underlying the necessity for Geriatric Oncology to develop.

Cancer has become a chronic disease thanks to advances in treatment. As patients suffer from a chronic condition, it appears that older adults with cancer could benefit from educational approaches and especially Therapeutic Patient Education (TPE). TPE is a basic, lasting component of patient management, according to the World Health Organization definition (WHO) (4). It aims at enabling people with chronic conditions to manage their illness and to cope with it in daily living, in partnership with health care professionals. TPE helps patients and their relatives acquire or maintain skills of self-management, through a patient-centred approach. By the mean of the specific methods and tools, tailored to the patient's need, organised educational activities are planned by a multidisciplinary team: physicians, nurses, dieticians, pharmacists, physiotherapists, ergotherapists, psychiatrists/psychologists, social workers, occupational health specialists, chiropodists and other professionals (specialists in education, health insurance specialists, hospital

administrators, school health educators and others). The components of TPE are patient-centred

communication tools (active listening, empathy and motivational interview), pedagogical methods (participative learning, brainstorming, roundtable and role-play case studies) and educational tools (audio, video, web-based programs, e-learning, booklets etc.) (WHO). The format of any TPE program includes individual and/or group sessions designed to provide information on the disease but also to share experience and knowledge. TPE is finally a continuous process, integrated into health care designed to help patients and their families live with a chronic condition, adhere to treatment and to limit the complications and consequences of the illness on their quality of life.

TPE has shown efficacy in the treatment of chronic pathologies, such as asthma (5), diabetes (6), psychiatric diseases (7) (8), or obesity (9). Recently, this approach became an important component in the management of patients with Alzheimer's disease and their caregivers (10). In oncology it is a fast-growing tool, and its interest is all the more important with ambulatory care taking the lead, given the appearance of numerous oral treatment options (11).

TPE appears to us as a key element in the management of older adults patients with cancer; indeed, if studies have shown that TPE has a positive impact on adherence (12), quality of life and pain (13) in adults suffering from chronic illness, it has also been shown that older adults could benefit from it (14). Moreover, in the geriatric literature and clinical routine geriatrics, we know that an educational component is needed in any intervention designed to limit or avoid geriatric syndromes such as falls (15, 16), frailty (17), malnutrition (18) and loss of autonomy in general (19). Considering these two facts, it can be envisaged that preventing those geriatric syndromes2 could also be targeted outcomes for educational interventions in older adults with cancer. In the specific and heterogeneous population of older adults with cancer, the therapeutic educational sessions's content must be tailored to, on the one hand, the disease (type of cancer), and on the other hand, the physiological or pathological age-related changes (mainly sensory and cognitive impairments) (20, 21, 22).

### Review objective

We chose to realise a systematic review designed to search for evidence of the effectiveness of therapeutic patient education interventions in older adults with cancer on physical and mental health.

## Materials and methods

### Inclusion criteria

#### Types of participants

This review considered studies that enrolled older patients, with an average age greater than 65 years old, of any gender and ethnicity, diagnosed with any form of cancer and receiving any treatments.

#### Types of interventions

This review considered studies in which the interventions included a therapeutic patient education aspect. Therapeutic patient education is rarely studied in itself in older adults with cancer, so we decided to consider any intervention with an educational component.

#### Comparator

We included studies in which the control group received information through usual care or usual education but not through a standardised multidisciplinary TPE method.

#### Types of outcomes

This review considered studies that included any outcome. We first envisage to study observance and quality of life, at the first step in our preliminary research, but finally chose to consider works studying any outcome.

#### Types of studies

This review considered experimental studies: randomised controlled trials. Other research designs such as quasi-experimental, before and after studies, prospective and retrospective studies, cohort studies, pilot studies and feasibility studies were also included.

### Exclusion criteria

This review excluded studies concerning subjects under 65 years old and non-educative interventions. We excluded qualitative studies and those published before 1990.

### Search Strategy

We analysed articles in English and French published between 1990 and July 2016. Several international databases were searched with identified keywords (Appendix 1): Medline, Cochrane Library, Web of Science and PsycINFO. A research of the grey literature was also conducted in Therapeutic Education and Geriatric Oncology journals.

One of the investigators is a Geriatrician. This research was conducted with the help of the primary care and family medicine department of the Toulouse University Hospital.

The second investigator is a Geriatrician too, who belongs to the Epidemiology and Public Health Department of the Faculty of Medicine of Toulouse. The research was conducted using identified keywords and index terms across all the included databases.

**Figure 1:**
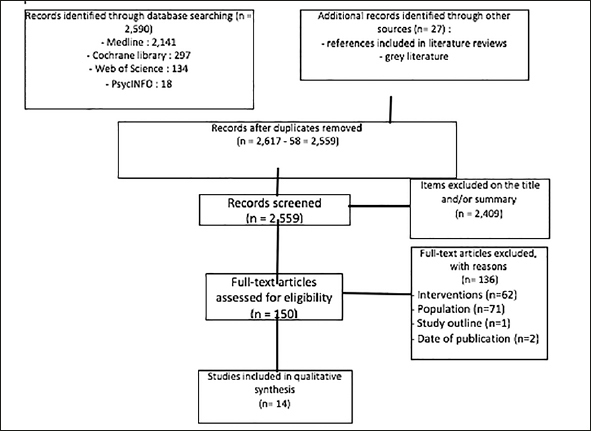
Flow Chart

The first screening found 2,617 articles. After reading the title and the abstract, 150 were eligible to be reviewed. Among them, which we read through, fourteen finally met the inclusion criteria. The selection process is presented in the flow-chart (Figure 1).

The articles included in this review were assessed independently by the two investigators, who reviewed the abstracts, read and selected the full texts independently.

### 2.4 Method of the review

#### Methodology quality

We assessed the methodological quality of each study included with validated scales:

• for randomised controlled trials: the CONSORT checklist (23) to analyse the quality of the report and Jadad score (24) to analyse the methodology of the study,
• for non-randomised studies: the STROBE checklist (25) to analyse the quality of the report and Newcastle-Ottawa criteria (26) to analyse the methodology of the study.

#### Data extraction

Data from the studies were extracted by two independent investigators. The data extracted included the title, authors, country and year of publication, and details about the study scheme, population (age and type of cancer), interventions, study methods, primary and secondary endpoints and the main outcomes.

#### Data synthesis

Due to the clinical and methodological heterogeneity between the included studies, a meta-analysis was not possible.

## Results

A total of fourteen articles were analysed in this literature review (Table 1). Among these fourteen articles, six were randomised controlled trials, three quasi-experimental studies, one prospective study, one cohort study, two pilot studies, and one feasibility study. They were conducted in several countries, with the majority in the United States (seven), Australia (two), Sweden (one), the Netherlands (two), Singapore (one) and Italy (one). They were published between 1993 and 2014.

**Table 1:** Main results of studies in geriatric population

**Reference, Year, Country**	**Study design**	**Population**	**Intervention**	**Outcome measures**	**Results**
(27) 2010 Australia	Randomised controlled trial	- 571 patients (88 % of eligible patients). - Mean age 65.3/64.2/63.9 years in the 3 arms. - Men with prostate or colorectal cancers, newly diagnosed.	- Type of intervention : Phone intervention (10 topics: cancer diagnosis, treatment/management issues, what to expect from surgery, management of side effects, communication with the specialist, partner/family issues, psychological/emotional and communication concerns, cancer language, diet and nutrition, other support services). - Performed : by trained cancer nurses - Duration : 12 months - Design : 3 study arms block randomised : * Active Referral-4 outgoing calls at 1 week, 6 weeks, 3 months and 6 months post-diagnosis. * Active Referral-1 outgoing call at 1 week of diagnosis * Passive referral (group control): usual care and contact at the participant's initiative - Block randomisation was used to reduce the changeover, demands on specialists. Specialists were randomised into one of the three study arms via computer generated random numbers.	- Primary outcome : Cancer specific distress (scale developed for breast cancer patients), anxiety and depression (HADS: hospital anxiety and depression scale) (28).	- There was no difference in mean levels of depression or cancer-specific distress between the three study arms. - Mean anxiety levels among the Active-Referral-1 outcall arm were significantly lower than for the Active-Referral-4 outcalls arm (5,1 vs 6,5 ; p=0,002).
(29) 2007 USA	Randomised, stratified, two-group, controlled clinical trial	- 103 patients (35,9 % of women) - Mean age of 72.4/71.4 in Geriatric population and 53.5/53.9 in non-Geriatric population. - Advanced cancer treated with radiotherapy.	- Type of intervention : Verbal instruction. Cognitive, emotional, physical, social, spiritual programs (8 sessions of 90 minutes, within the first 4 weeks) : conditioning exercises followed by educational instruction on symptom management, spiritual guidance, information on financial resources, advanced directives, cognitive behavioral training for coping with cancer, open discussion and support, relaxation exercises). - Performed : by a psychiatrist or psychologist and nurse, physical therapist, chaplain, or social worker - Duration : 27 weeks - Design : 2 groups randomised and stratified : * intervention group * standard care group	- In the geriatric group only, significantly higher overall QOL scores in the intervention group at week 4 (79,3 vs 62,9, p=0,0461). - The particular domains of QOL on which the geriatric intervention group scored significantly higher compared to other groups included spiritual well-being at week 4 and emotional well-being at week 8.	
(32) 1999 Sweden	Randomised controlled trial	- 527 patients included, 381 randomised (60 % of women). - Mean age of 65. - Breast, colorectal and prostate cancers.	- Type of intervention : Phone intervention. Regular follow-up contacts, routines to improve general practitioners' and home care nurses' possibilities to support and monitor patients. - Performed : by a home care nurse - Duration : 6 months - Design : 4 groups randomised : * Intervention group (Intensified Primary Care, IPC) with 2 subgroups: Individual Support (IS) with psychological and dietician support OR a combination of individual support and rehabilitation (ISR). * Control group with 2 subgroups: Rehabilitation (R) with group intervention 3 months after diagnosis OR Standard Care (SC).	- Primary outcome : Frequency of follow-up contacts with the home care nurse at 6 months and patient satisfaction with the follow-up contacts (18-item questionnaire). - Secondary outcomes : Anxiety and depression (HADS).	- Significant lower number of contact in the control group than in the intervention group during the first 6 months (74 % vs <30 % ; p<0,001). - 6 % of control patients and 43 % of intervention group reported continuing contact with the home care nurse at 6 months (p <0,001). - The majority of IPC patients evaluated the intervention positively. - In the multivariate analysis, anxiety contributed to the model in the likelihood ratio test although it was no significant.
(33) 2006 USA	Randomised controlled trial	- 231 patients registered, 192 patients randomised and 131 completed the study (47 % of women). - Mean age of 73 in TM + EM group and 74 in EM group (> 65 years only). - Advanced breast, prostate or colon cancers.	- Type of intervention group : Phone intervention and educational materials. TM (telephone monitoring) + EM (educational materials), 1 call per month. - Performed : by centralised, trained telephone monitors - Duration : 6 months + 3 months of observation - Design : 2 groups randomised : * Intervention group * Control group: EM only.	- Primary outcomes : physical problem, psychological distress, social support distress (EORTC-QLQ-C30 (34), Geriatric Depression Scale (35), HADS, Medical Outcomes Study Social Support Survey (36), Utilisation of Mental Health and Psychological Services instrument (37), Geriatric Schedule of Recent Experience (GSRE)(38), Physical Health subscale of the Older American Resources and Services Questionnaire (39). - Secondary outcomes : Patient satisfaction (Research Program BOMC test) (40).	- Significantly lower anxiety and depression in the intervention group than in the control group at 6 months (HADS total score, 6,01 vs 8,20, p < 0,0001 ; HADS depression subscale, 3,20 vs 4,08, p=0,0004 ; HADS anxiety subscale, 2,81 vs 3,25, p<0,0001) - No significant difference for the other outcomes measured.
(41) 1993 USA	Randomised controlled trial - Mean age of 66 (> 60 years only).	- 40 patients. - The major cancer diagnoses were prostate, colon, lung and breast.	- Type of intervention : Verbal instruction and educational materials. The three-part intervention at home (Part I included general information about pain. Part II focused on pharmacologic interventions. Part III included information about nondrug pain management and demonstration of nondrug pain), written patient education booklet, two audiocassette tapes and written instructions for each of 19 nondrug interventions. Management techniques. - Performed : by a nurse - Duration : four weeks after the instruction day - Design : 2 groups randomised : * intervention group * control group : usual care Subjects were stratified as either elderly (60-75 years) or oldest (>75 years).	- Primary outcomes : Quality of life (quality of life tool), knowledge and attitudes of the caregiver and patient in managing pain (patient pain questionnaire), compliance with the drug and nondrug interventions and perceived effectiveness (self-care log (42)), mood (profile of mood states (43)). - Secondary outcomes : caregiver burden.	- Significant decrease in pain intensity (p=0,05), perception of pain severity (p=0,001), fear of addiction (p=0,02), anxiety (p=0,05) and increase of the use of pain medications (p=0,01). - Significant improvement in sleep (p=0,03) and rise of knowledge levels (use of medications on schedule rather than an as-needed basis) (p=0,007). - Family caregivers benefit from this education program.
(44) 2011 The Netherlands	Randomised, controlled pre- and post-test design	- 343 eligible patients and 210 patients participated (34,8 % of women). - Mean age of 72.2 (> 65 years only). - Patients receiving chemotherapy for the first time or for the first time in 5 years.	- Type of intervention : Verbal instruction and educational materials. A web-enabled video feedback, a one-day training session, a follow-up meeting and a patient booklet including a Question Prompt Sheet. The information developed were classified in 17 topics, ranging from treatment-related topics and dealing with side effects to emotional topics, coping with illness and sexuality. - Performed : by nurses - Duration : six weeks after the training day - Design : 2 groups randomised by having lots drawn from a sealed container by an independent person : * Intervention group * Control group: usual education A randomised pre- and post-test design was used. The video-observations were conducted by blinded observers.	- Primary outcome : Recall of information measured using the “Netherlands Patient Information recall Questionnaire” (NPIRQ). - Secondary outcomes : Quality of communication was measured by the QUOTE chemo-Performance, frailty was measured using the Groningen Frailty Indicator (45), amount of questions.	- Significant decrease in the number of items discussed (total change score — 9,87, p < 0,001). - Significant positive intervention effect in “discussing realistic expectations” (total change score 0,65, p< 0,01). - Limited intervention effect on recall of information with a significant pre-/post-change in proportion recall of two categories of recommendations : “hygiene” (total change score 24,79, p<0,05), and “side effects that have to be reported to the hospital” (total change score 15,86, p<0,05).
(46) 1999 USA	Quasi-experimental study	- 85 patients (1 % of women). - Mean age of 65. - Patients with newly diagnosed cancer who were receiving chemotherapy.	- Type of intervention : Educational materials. The educational materials were provided in a folder that was labelled “Patient Information Packet” (PIP). There are 6 items: call for help in case of serious complications, chemotherapy side effects, resource sheet (phone numbers of services needed), support group, information about the patients' specific malignancy and a fact sheet about the chemotherapeutic agents being administered. - Performed : by the oncology pharmacist - Duration : evaluation one month after the distribution of the PIP - Design : The PIP was distributed by the pharmacist who explain the contents of the PIP. One month after, he evaluated the usefulness of the material by interviewing each patient.	- Primary outcome : Usefulness of the materials (how often the materials has been read and utilised and whether the patient perceived the materials to be helpful).	- 82 of the 85 patients had utilised the materials in the PIP. 44 patients had read the materials once, 32 patients read them twice, 10 patients had read them three times, and 6 patients read them four or more times. - The PIP had helped 13 patients decide to call the hospital and had helped 16 patients decide whom to call. - The patients said the materials was good, informative and easy to read.
(47) 1999 USA	Quasi-experimental pre/post-test design - Mean age of 76/77 by groups (> 65 years only).	- 60 patients randomised, 53 patients completed the study, 36 patients analysed (63,9% of women). - Prostate, bladder, colon, lung, breast cancers, lymphoma.	- Type of intervention : Educational materials. A booklet “Managing Cancer Pain” and a 14-minute-video. Methods of pain management, including non-drug interventions, were discussed as well as types of medications, timing of administration, routes of administration, benefits and risks, side effects and management. - Performed : by physician or office staff - Duration : contact two weeks after the pre-test - Design : 2 groups randomised (each subject draw a folded slip of paper marked with « E » or « C » from a box containing an equal number of both designations) : * Experimental group * Control group	- Primary outcome : Pain intensity measured by the Visual Analog Scale (VAS) (48).	- Significant decrease of pain intensity in the intervention group (F=5,8, p=0,021).
(49) 2008 The Netherlands	Quasi-experimental study	- 83 participating patients whose 69 patients studied (30,4 % of women). - Mean age of 71.8 (> 65 years only). - Chemotherapy treatment.	- Type of intervention : Verbal instruction. An education session, which was recorded by video. The questions covered two main domains: information about treatment and recommendations on coping with potential side effects. - Performed : by a nurse - Duration : one consultation - Design : Patients come to a consultation with a nurse with videorecording. Immediately after the consultation, patients were asked to complete a questionnaire.	- Primary outcome : Recall of information measuring by “The Netherlands Patient Information Recall Questionnaire” .	- Older patients had difficulty remembering items. - The patients recalled only 23.2 % of the recommendations given on handling side effects as measured with open-ended questions.
(50) 2013 Singapore	Prospective study	- 118 patients (44,1 % of women). - Mean age of 71.7 (> 65 years only).	- Type of intervention : Phone intervention. Medication therapy management (MTM) is a service group that optimise therapeutic outcomes for individual patients: * assessments of the patient's health status to identify, resolve, and prevent medication-related problems; * formulating a medication treatment plan; * selecting, initiating, modifying, or administering medications; * providing verbal education to enhance patient adherence; * documenting the care delivered and communicating essential information to the patient's other primary care providers; * coordinating and integrating medication therapy management services; * monitoring and evaluating the patients' responses to therapy, including safety and effectiveness. - Performed : by pharmacists - Design : Prospective study conducted via direct interview with patients/caregivers. Pre- and post-service patient satisfaction surveys were conducted before and after MTM.	- Primary outcome : identification of drug-related problems (DRP), classification of the DRP in 9 categories. - Secondary outcomes : Effectiveness of MTM service in resolving DRP, clinical significance of pharmacist interventions, patients' satisfaction level using a patient satisfaction survey.	- 361 DRP have been identified and resolved. The most common DRP were drug interactions, adverse effects, non-adherence. - 44 interventions were performed by pharmacists and 40 cases were accepted by physicians. - Almost 2/3 interventions were deemed significant by the judges. - There was statistically significant improvement in patientsâ€™ satisfaction level after the service was provided (p<0,001).
(51) 2014 Australia	Cohort study	- 859 patients. - Mean age of 71 +/-8 years. - Prostate cancer.	- Type of intervention : Verbal instruction. * Face to face exercise group : 10-week exercise program (2 group sessions per week supervised by an accredited exercise physiologist) followed by tailored at home exercises for 6 months, home based exercise program for 6 months and support program. * At home exercise group : home-based exercise program for 6 months (coach calls, exercise manual, DVD). * Man plan support program : education on low-intensity exercise, diet and psychosexual functions. - Performed : by an accredited exercise physiologist - Duration : 6 months and access to phone line during 2 years - Design : 3 groups according to patient preference, medical comorbidities and fitness level.	- Primary outcomes : Body composition variables cardiovascular and cardiorespiratory fitness variables, strength variables. - Secondary outcome : Patient satisfaction.	- Significant reduction in mean waist (p<0,0001) and hip circumference (p=0,015), blood pressure (p=0,0044 for systolic blood pressure ; p<0,0001 for diastolic blood pressure), mean time for completing the 400-m walk (p<0,0001) and improvements in ability to undertake resistance training exercises (p<0,0001). - No significant modification of weight, BMI (Body Mass Index) and heart rate. - High satisfaction, recruitment, and compliance.
(21) 2010 USA	Pilot Study	- 21 patients (52,4 % of women). - Patients >65 years. - Breast, pancreatic, head and neck, lung and colon cancers, lymphoma, myelodysplasia.	- Type of intervention : Verbal instruction and educational materials. The intervention associated teaching sessions (conducted by a nurse, who discussed the educational materials) and educational materials (brochures). - Performed : by trained nurses - Duration : initial teaching session and survey at 3 or 4 weeks - Design : Brochures were offered to the patient. Teaching sessions were conducted by a nurse. The survey was administered by one trained nurse.	- Primary outcomes : Patient perceptions of the clarity and quality of the materials and patient retention of key information delivered in the teaching session (oral survey).	- Positive feedback on the materials. - The patients generally were able to recall and provide examples of how to manage the most important side effects of chemotherapy.
(52) 1990 USA	Pilot study	- 21 patients (81 % of women). - Mean age of 80 (> 65 years only).	- Type of intervention : Verbal instruction and educational materials. Modifications of the conventional cancer education program: * presenting materials (slower pace, careful organization of the presentation in a logical and meaningful way, adapted slides, simple examples, concrete illustrations, encouraging familiar aids, group sized limited to 15, informal atmosphere…). * education program (discussion about cancer risk with age and importance of early diagnosis, concrete examples, discussion about the current cancer screening guidelines…). - Performed : by a general nurse practitioner - Duration : one teaching session and post-test 1 week later - Design : patients were randomly assigned to one of the three group : * intervention group : modified cancer education program * conventional cancer education program (videos) * educational program on nutrition by a nurse educator	- Primary outcome : Cancer knowledge and beliefs measured by the Health Belief Model of Rosenstock and Becker (53).	- Significant increase in cancer knowledge on the post-test in the intervention group (t(11) = −2,53, p< 0,05).
(54) 2014 Italy	Feasibility study	- 81 patients (54 % of women). - Median age of 68. - Breast, colon-rectum, stomach, liver, lung, kidney, pancreas, stromal, small bowel and anus cancers.	- Type of intervention: Verbal instruction and phone intervention. * initial training about: how to take the oral medication, toxicity profile and the recognition of side effects, instructions for their management and the actions to be taken in case of discontinuation of therapy… * diary including a calendar to check off pill consumption for each day and a specific form to collect a self-report of toxicity. * Patients were monitored during the first and second cycles of oral therapy, by phone calls on days 7 and 14. - Performed : by a nurse - Duration : monitoring for two weeks - Design : after a medical visit the patient received training by the nurse who showed the patient how to correctly take the oral medication. A questionnaire was administered before and after training by the nurse.	- Primary outcome : Quality of care evaluated by a questionnaire with specific items concerning the level of comprehension. - Secondary outcomes : The nurse collected the diary and asked the patient to describe and specify every symptom, and graded the toxicity according to NCI-CT-CAE3.0(55).	- The intervention resulted in an increased proportion of patients having received correct information related to treatment, with a level of confidence rising to more than 90 % for all items considered. - The diary proved a valid tool for patients. - This model proved practicable and accepted by patients.

There is great heterogeneity in the populations studied. Regarding our targeted population, it appears that older adults (over 65) were identified and specifically studied in only seven studies (33, 44, 47, 49, 50, 21, 52). In other studies, older participants were pooled with a general adult population. In one study, there was a comparison between a geriatric and a non-geriatric group (29).

The types of cancer studied are also diverse. The most prevalent were colorectal, prostate, breast and lung cancers, although there was also bladder, pancreatic, stomach, liver, kidney and small intestine cancers, lymphomas and myelodysplasias. Cancer stages varied according to the studies, as well as the treatments (chemotherapy and radiotherapy) received.

The interventions types were numerous. The vast majority of interventions were multi-dimensional and included an educational aspect, but were not exclusively educational or pedagogic.

The interventions' follow up were also various. Patients were followed-up by phone (27, 32, 33, 50) or at home, through psychological support or via distribution of educational materials (33, 41, 44, 46, 47, 21, 52) in various forms (brochures, booklets, audio or video links to the Internet …). Educational interventions were mainly carried out through tools such as the telephone, video or the Internet. These interventions were not adapted to the specific learning capabilities of older adults except in one study (52).

Regarding the evaluation criteria, the most frequently assessed outcomes were anxiety, depression and psychological distress (27, 32, 33), as well as the patient's knowledge and understanding (41, 44, 49, 52, 54) and their satisfaction (33) (50, 51). Many other criteira were represented: usefulness of intervention (46, 21), pain (47), the overall quality of life (29, 41), drug toxicity (50), patient compliance (41), quality of communication (44), quality of the monitoring (32) and physical health (51).

The more frequently found significant positive results were observed on pain, anxiety and quality of life (three for anxiety, two for pain and two for quality of life). One study showed an improvement of the patient's depression (33) but another found no difference (27). Concerning the patient's level of information (and recall of information) a majority of the studies were positive, only one found difficulty in remembering information for patients (49).

Thus, only one study (52) offered a suitably adapted program of therapeutic education (on the presentation of the educational material and the content of the information) to a geriatric population with an average age of 80 years of age. This study shows an increase in knowledge about cancer after this intervention. However, it deals with patients who do not have cancer but whose aim is preventive health care of older adults with respect to cancer. In addition, this was a pilot study with only 21 enrolled patients and an average methodological quality.

The methodological quality of the included studies have been assessed by the validated scales : the Jadad scale (24) or the Newcastle-Ottawa quality assessment scale (26). The STROBE statement (25) or CONSORT checklist (23) have been used to estimate the quality of the study report. Tables 2 and 3 show summaries for each study, their detailed scores on the scales. In the randomised trials, blinding was impossible because of the type of interventions. Two of the randomised trials (27, 44) are of good methodological quality (Jadad score of 3).

**Table 2:** Methodological evaluation of randomised controlled trials

		**JADAD**		**CONSORT**
	**Randomisation (/2)**	**Blinding (/2)**	**An account of all patients (/1)**	
Livingston and al, 2010, « The psychological impact of a specialist referral and telephone intervention on male cancer patients: a randomised controlled trial » (27)	2 3/5	0	1	22/29 (75.9%)
Lapid and al, 2007, « Improving the quality of life of geriatric cancer patients with a structured multidisciplinary intervention: a randomized controlled trial » (29)	1 1/5	0	0	18/28 (64.3%)
Johansson and al, 1999, « Intensified primary cancer care: a randomized study of home care nurse contacts » (32)	1 2/5	0	1	15/28 (53.6%)
Kornblith and al, 2006, « Telephone monitoring of distress in patients aged 65 years or older with advanced stage cancer: a cancer and leukemia group B study » (33)	1 2/5	0	1	20/28 (71.4%)
Ferrell and al, 1993, « Development and implementation of a pain education program » (41)	1 1/5	0	0	5/29 (17.2%)
Van Weert and al, 2011, « Effects of communication skills training and a Question Prompt Sheet to improve communication with older cancer patients: a randomized controlled trial » (44)	2 3/5	0	1	22/28 (78.6%)

**Table 3:** Methodological evaluation of non-randomised controlled trials

		**Newcastle-Ottawa**		**STROBE**
	**Selection (/4)**	**Comparibility (/2)**	**Outcome (/3)**	
Jazieh and al, 1999, « Development of a patient information packet for veterans with cancer receiving chemotherapy » (46)	3 4/9 (44.4%)	0	1	11/28 (39.3%)
Clotfelter and al, 1999, « The effect of an educational intervention on decreasing pain intensity in elderly people with cancer » (47)	4 9/9 (100%)	2	3	19/28 (67.9%)
Jansen and al, 2008, « Recall in older cancer patients: measuring memory for medical information » (49)	3 5/9 (55.6%)	0	2	19/28 (67.9%)
Yeoh and al, 2013, « The impact of medication therapy management in older oncology patients » (50)	3 5/9 (55.6%)	0	2	18/28 (64.3%)
Beydoun and al, 2014, « Prospective study of exercise intervention in prostate cancer patients on androgen deprivation therapy » (51)	4 8/9 (88.9%)	1	3	21/28 (75%)
Rigdon and al, 2010, « Development of patient education for older adults receiving chemotherapy » (21)	3 4/9 (44.4%)	0	1	12/28 (42.9%)
Barnes and al, 1990, « A modified cancer education program. Effect on cancer knowledge and beliefs of the elderly » (52)	3 5/9 (55.6%)	0	2	19/28 (67.9%)
Cirillo and al, 2014, « Management of oral anticancer drugs: feasibility and patient approval of a specific monitoring program » (54)	3 5/9 (55.6%)	0	2	15/28 (53.6%)

Among the non-randomised trials (47, 51), two studies are of good methodological quality (Newcastle-Ottawa score > 75%).

Given the heterogeneity of the studies, we were not able to perform a meta-analysis.

## Discussion and Conclusion

### Discussion

Only fourteen studies were included in this literature review studying educational interventions in older patients with cancer. The results of these studies are quite positive overall. Those interventions seem to provide positive effects on health outcomes but not only (knowledge and quality of life). However, none of these articles studied the effectiveness of a TPE specifically tailored for older patients with cancer (over 65 years of age). There is currently very little data in the literature on the effectiveness of Therapeutic Education in this population.

If data on patient education in geriatric oncology is poor, we realise that data was also lacking in adults under 65 years of age, through this literature review (56). Indeed, a systematic literature review performed in 2015 by a US team (56) found only two articles on the effectiveness of therapeutic education for adult patients regarding their compliance/observance with oral cancer treatment (average age 56 years (57) and 59.85 years old (58)) with cancer in an outpatient environment, between 1953 and 2014. These two studies had small-sized samples and their methodology was from weak to moderate. Therefore, the conclusion of this literature review is that further studies are needed to demonstrate that patient education can improve compliance with cancer treatments and their health outcomes. This is in accordance with our findings; data is limited in adult populations, but even more in older adults, virtually non-existent.

Our study raises this question: why Therapeutic Patient Education studies are so few in older populations despite the fact that TPE is recommended in chronic conditions and is expanding in Geriatrics and in Oncology? In a general manner, older patients with cancer are underrepresented in clinical trials. A 2012 article (59) showed that inclusion diminishes with age in these types of studies. The authors surmised that a decline in the functional reserve, increased comorbid conditions, concomitant medication use, lack of social/home support and decreased access among other factors contributed to poor enrolment among older adults. To remedy this situation, studies addressing older subjects need to take into account those specificities and need to be designed to gauge the weight of these specificities in this population. For example, there is a need to adapt interventions to the specific learning capabilities of older adults as mentioned by Barnes et al. (52). There is also a need to take into account the health care professionals; skills and needs in older adults with cancer management. On the one side, health care professionals feel their formation is lacking to address to accompany older subjects with cancer, which is a time-consuming activity for which one has to be committed. On the other side, they feel as though they are already giving out enough information, but it is not equivalent to using specific tools and pedagogical methods of education (60).

Thus, TPE, included in an integrated care management strategy, could provide benefits in terms of mental and physical health for the patient, their relatives but also in terms of health care system utilisation (avoid inappropriate admission, iatrogenia). It could increase the patient's observance to the treatment, increasing the latter's health outcomes, as it has been proven in other chronic diseases such as diabetes (6) or asthma (5). It could also decrease, and that is a major topic in Geriatric Oncology, the toxicity of chemotherapy by decreasing overuse and even reduce the misuse of care resources, in particular, hospitalisation.

Finally, the increase in the patient's knowledge would allow them to be more involved in their care and enhance their role in the decision-making process.

#### Strengths

The main strength of this study is the innovative character of the approach since it is the first literature review on this topic in this population. In fact, this is a current topic, with the gradual increase in cancer prevalence in the patient population over 65 years of age.

#### Limitations

A major limitation of this study is the heterogeneity of the studies included, particularly because of their different study designs; their interventions are not fully comparable and their methodological quality is variable. The differences between the populations, health systems, the type of intervention, outcomes and methodological quality compound this heterogeneity. The fourteen studies included mostly had a small patient sample, which limits their ability to show a significant difference and makes it difficult to extract generalisations.

These heterogeneous results are due to the lack of scientific data on the efficacy of therapeutic patient education in older adults. We had to open the field of research to include any type of educational intervention in this population to show that it might indeed be a feasible and useful proposition. We feel that TPE including caregivers could improve parameters such as quality of life, compliance and pain management, amongst others. Our key finding is that data is missing regarding this subject in the scientific literature.

Geriatric oncology is developing, as well as the use of TPE as part of the care plan for these patients. Tailored TPE programs for older patients with cancer are implemented. Studies on this topic are becoming more numerous since six out of the fourteen selected articles were published after 2010.

There are several perspectives on TPE in geriatric oncology. We can surmise that, in the future, programs will be partly carried out by information and communication technologies. Indeed, we could imagine that, with the development of telemedicine, part of the educational approach could be carried out remotely in the form of online courses or discussion forum online with health care providers, for example.

## Conclusion

There is a lack of data on TPE in the field of geriatric oncology. The effectiveness of a therapeutic education program for older adult cancer patients must be studied because of the efficacy of TPE in chronic conditions, the prevalence of cancer in older adults and the global ageing of the population. TPE could increase treatment compliance/observance, decrease side effects, improve health outcomes and have a positive effect on the quality of life of these patients and their relatives. Further studies, and especially studies of high methodological quality and level of evidence, are needed to assess the effectiveness of TPE in older adults with cancer.

## Electronic supplementary material

Appendix 1 : Search criteria used on the databases

## References

[1] Cancer Incidence in Five Continents Vol. X. Disponible sur: http://www.iarc.fr/en/publications/pdfs-online/epi/sp164/

[2] Ã‰pidÃ©miologie des cancers chez les patients de 65 ans et plus â€” OncogÃ©riatrie | Institut National Du Cancer. Disponible sur: http://www.e-cancer.fr/Professionnels-de-sante/L-organisation-de-l-offre-de-soins/Oncogeriatrie/Epidemiologie

[3] Institut national du cancer. Les cancers en France â€” Edition 2014. Disponible sur: http://www.unicancer.fr/sites/default/files/Les%20cancers%20en%20France%20-%20Edition%202014%20-%20V5.pdf

[4] Therapeutic patient education: continuing education programmes for health care providers in the field of prevention of chronic diseases: report of a WHO working group. Disponible sur: http://apps.who.int/iris/handle/10665/108151

[5] Horn IB, Mitchell SJ, Gillespie CW, Burke KM, Godoy L, Teach SJ (nov 2014). Randomized trial of a health communication intervention for parents of children with asthma. J Asthma Off J Assoc Care Asthma..

[6] Golay A, Lagger G, Chambouleyron M, Carrard I, Lasserre-Moutet A (2008). Therapeutic education of diabetic patients. Diabetes Metab Res Rev..

[7] Pitschel-Walz G, BÃ¤uml J, Bender W, Engel RR, Wagner M, Kissling W (2006). Psychoeducation and compliance in the treatment of schizophrenia: results of the Munich Psychosis Information Project Study. J Clin Psychiatry..

[8] Shimodera S, Furukawa TA, Mino Y, Shimazu K, Nishida A, Inoue S (2012). Cost-effectiveness of family psychoeducation to prevent relapse in major depression: results from a randomized controlled trial. BMC Psychiatry..

[9] Snethen JA, Broome ME, Cashin SE (2006). Effective weight loss for overweight children: a meta-analysis of intervention studies. J Pediatr Nurs..

[10] Ã‰ducation thÃ©rapeutique dans la maladie dâ€™Alzheimer. Disponible sur: https://link.springer.com/article/10.1007%2Fs12612-014-0430-6

[11] UNICANCER â€” Quelle prise en charge des cancers en 2020 ? Disponible sur: http://www.unicancer.fr/patients/quelle-prise-charge-cancers-2020

[12] Foulon V, SchÃ¶ffski P, Wolter P (2011). Patient adherence to oral anticancer drugs: an emerging issue in modern oncology. Acta Clin Belg..

[13] Ferrell BR, Rivera LM (1997). Cancer pain education for patients. Semin Oncol Nurs..

[14] Pariel S, BoissiÃ¨res A, Delamare D, Belmin J [ (2013). Patient education in geriatrics: which specificities?. Presse Medicale Paris Fr 1983.

[15] Moylan KC, Binder EF (juin 2007). Falls in older adults: risk assessment, management and prevention. Am J Med..

[16] Clemson L, Cumming RG, Kendig H, Swann M, Heard R, Taylor K (2004). The effectiveness of a community-based program for reducing the incidence of falls in the elderly: a randomized trial. J Am Geriatr Soc..

[17] Leveille SG, Wagner EH, Davis C, Grothaus L, Wallace J, LoGerfo M (1998). Preventing disability and managing chronic illness in frail older adults: a randomized trial of a community-based partnership with primary care. J Am Geriatr Soc..

[18] Bandayrel K, Wong S (2011). Systematic literature review of randomized control trials assessing the effectiveness of nutrition interventions in community-dwelling older adults. J Nutr Educ Behav..

[19] Gill TM, Baker DI, Gottschalk M, Peduzzi PN, Allore H, Byers A (oct 2002). A program to prevent functional decline in physically frail, elderly persons who live at home. N Engl J Med. 3.

[20] Weinrich SP, Boyd M, Nussbaum J (1989). Continuing educationâ€”adapting strategies to teach the elderly. J Gerontol Nurs..

[21] Rigdon AS (2010). Development of patient education for older adults receiving chemotherapy. Clin J Oncol Nurs..

[22] Alywahby NF (dec 1989). Principles of teaching for individual learning of older adults. Rehabil Nurs Off J Assoc Rehabil Nurses..

[23] The CONSORT statement: revised recommendations for improving the quality of reports of parallel-group randomised trials. Disponible sur: https://www.sciencedirect.com/science/article/pii/S0140673600043373 10.1007/s00784-002-0188-x 12673431

[24] Assessing the Quality of Randomized Trials â€” Controlled Clinical Trials. Disponible sur: https://www.contemporaryclinicaltrials.com/article/S0197-2456(99)00026-4/abstract

[25] Elm E von, Altman DG, Egger M, Pocock SJ, GÃ¸tzsche PC, Vandenbroucke JP (2007). The Strengthening the Reporting of Observational Studies in Epidemiology (STROBE) Statement: Guidelines for Reporting Observational Studies. PLOS Med..

[26] Stang A (2010). Critical evaluation of the Newcastle-Ottawa scale for the assessment of the quality of nonrandomized studies in meta-analyses. Eur J Epidemiol..

[27] Livingston PM, White VM, Hayman J, Maunsell E, Dunn SM, Hill D (2010). The psychological impact of a specialist referral and telephone intervention on male cancer patients: a randomised controlled trial. Psychooncology..

[28] Zigmond AS, Snaith RP (1983). The hospital anxiety and depression scale. Acta Psychiatr Scand..

[29] Lapid MI, Rummans TA, Brown PD, Frost MH, Johnson ME, Huschka MM (2007). Improving the quality of life of geriatric cancer patients with a structured multidisciplinary intervention: a randomized controlled trial. Palliat Support Care..

[32] Johansson B, Berglund G, Glimelius B, Holmberg L, SjÃ¶dÃ©n PO (1999). Intensified primary cancer care: a randomized study of home care nurse contacts. J Adv Nurs..

[33] Kornblith AB, Dowell JM, Herndon JE, Engelman BJ, Bauer-Wu S, Small EJ (2006). Telephone monitoring of distress in patients aged 65 years or older with advanced stage cancer: a cancer and leukemia group B study. Cancer.

[34] Aaronson NK, Ahmedzai S, Bergman B, Bullinger M, Cull A, Duez NJ (1993). The European Organization for Research and Treatment of Cancer QLQ-C30: a quality-of-life instrument for use in international clinical trials in oncology. J Natl Cancer Inst..

[35] Yesavage JA, Brink TL, Rose TL, Lum O, Huang V, Adey M (1982). Development and validation of a geriatric depression screening scale: a preliminary report. J Psychiatr Res..

[36] Sherbourne CD, Stewart AL (1991). The MOS social support survey. Soc Sci Med 1982.

[37] Siegel K, Raveis VH, Mor V, Houts P (juill 1991). The relationship of spousal caregiver burden to patient disease and treatment-related conditions. Ann Oncol Off J Eur Soc Med Oncol..

[38] Amster LE, Krauss HH (1974). The relationship between life crises and mental deterioration in old age. Int J Aging Hum Dev..

[39] George LK, Fillenbaum GG (1985). OARS methodology. A decade of experience in geriatric assessment. J Am Geriatr Soc..

[40] Katzman R, Brown T, Fuld P, Peck A, Schechter R, Schimmel H (1983). Validation of a short Orientation-Memory-Concentration Test of cognitive impairment. Am J Psychiatry..

[41] Ferrell BR, Rhiner M, Ferrell BA (1993). Development and implementation of a pain education program. Cancer.

[42] Dodd MJ (1984). Measuring informational intervention for chemotherapy knowledge and self-care behavior. Res Nurs Health..

[43] McNair DM, Lorr M, Droppleman LF (1971). Profiles of mood states.

[44] van Weert JCM, Jansen J, Spreeuwenberg PMM, van Dulmen S, Bensing JM (2011). Effects of communication skills training and a Question Prompt Sheet to improve communication with older cancer patients: a randomized controlled trial. Crit Rev Oncol Hematol..

[45] Old or frail: what tells us more? Disponible sur: https://www.ncbi.nlm.nih.gov/pubmed/15472162

[46] Jazieh AR, Brown D (1999). Development of a patient information packet for veterans with cancer receiving chemotherapy. J Cancer Educ Off J Am Assoc Cancer Educ..

[47] Clotfelter CE (1999). The effect of an educational intervention on decreasing pain intensity in elderly people with cancer. Oncol Nurs Forum..

[48] Grossman SA, Sheidler VR, McGuire DB, Geer C, Santor D, Piantadosi S (1992). A comparison of the Hopkins Pain Rating Instrument with standard visual analogue and verbal descriptor scales in patients with cancer pain. J Pain Symptom Manage..

[49] Jansen J, van Weert J, van der Meulen N, van DÃ¼lmen S, Heeren T, Bensing J (2008). Recall in older cancer patients: measuring memory for medical information. The Gerontologist..

[50] Yeoh TT, Si P, Chew L (mai 2013). The impact of medication therapy management in older oncology patients. Support Care Cancer Off J Multinatl Assoc Support Care Cancer..

[51] Beydoun N, Bucci JA, Chin YS, Spry N, Newton R, GalvÃ£o DA (2014). Prospective study of exercise intervention in prostate cancer patients on androgen deprivation therapy. J Med Imaging Radiat Oncol..

[52] Barnes S, Thomas A (1990). A modified cancer education program. Effect on cancer knowledge and beliefs of the elderly. Cancer Nurs..

[53] Rosenstock IM, Strecher VJ, Becker MH (1988). Social learning theory and the Health Belief Model. Health Educ Q..

[54] Cirillo M, Lunardi G, Coati F, Ciccarelli L, Alestra S, Mariotto M (2014). Management of oral anticancer drugs: feasibility and patient approval of a specific monitoring program. Tumori..

[56] Arthurs G, Simpson J, Brown A, Kyaw O, Shyrier S, Concert CM (2015). The effectiveness of therapeutic patient education on adherence to oral anti-cancer medicines in adult cancer patients in ambulatory care settings: a systematic review. JBI Database Syst Rev Implement Rep..

[57] Schneider SM, Adams DB, Gosselin T (2014). A tailored nurse coaching intervention for oral chemotherapy adherence. J Adv Pract Oncol..

[58] Simons S, Ringsdorf S, Braun M, Mey UJ, Schwindt PF, Ko YD (juill 2011). Enhancing adherence to capecitabine chemotherapy by means of multidisciplinary pharmaceutical care. Support Care Cancer Off J Multinatl Assoc Support Care Cancer..

[59] Scher KS, Hurria A (juin 2012). Under-representation of older adults in cancer registration trials: known problem, little progress. J Clin Oncol Off J Am Soc Clin Oncol. 10.

[60] Engel S, Reiter-JÃ¤schke A, Hofner B [ (2016). â€œEduKation demenz. Psychoeducative training program for relatives of people with dementia. Z Gerontol Geriatr..

