# Supplementary material for: Effectiveness of Therapeutic Patient Education Interventions for Older Adults with Cancer: A Systematic Review
Source: J Nutr Health Aging. 2020 May 20;24(7):772–82. doi: 10.1007/s12603-020-1395-3 (PMC12876787; doi:10.1007/s12603-020-1395-3)
Supplement: Supplementary file 1 — Appendix 1 : Search criteria used on the databases [file mmc1.doc]

**Appendix 1 :**  **Search criteria used on the databases**

| DATABASE | SEARCH CRITERIA |
| --- | --- |
| Medline | ("Patient Education as Topic"[MeSH] OR "Patient Education Handout"[Publication Type] OR "Physician-Patient Relations "[MeSH] OR "Program Development"[MeSH] OR "Self Care"[MeSH] OR (patient education) OR (patient counseling) OR (therapeutic patient education) OR (patient education program) OR (patient education intervention) OR (educational needs) OR (patient centered approach) OR (psychosocial intervention) OR (self management)) AND ("Aged”[MeSH] OR "Geriatrics"[MeSH] OR oncogeriatry OR (geriatric oncology) OR (cancer elderly) OR (older cancer patients)) AND ("Medical Oncology"[MeSH] OR "Neoplasms/drug therapy"[MeSH] OR "Neoplasms/education"[MeSH] OR "Neoplasms/therapeutic use"[MeSH] OR (newly diagnosed cancer)) |
| Cochrane Library | ("Patient Education as Topic"[MeSH] OR "Physician-Patient Relations "[MeSH] OR "Program Development"[MeSH] OR "Self Care"[MeSH] OR (patient education) OR (patient counseling) OR (therapeutic patient education) OR (patient education program) OR (educational needs) OR (patient centered approach) OR (patient education intervention) OR (self management)) AND ("Aged”[MeSH] OR "Geriatrics"[MeSH] OR (geriatric oncology) OR (cancer elderly) OR (older cancer patient)) AND ("Medical Oncology"[MeSH] OR "Neoplasms"[MeSH] OR (newly diagnosed cancer)) |
| Web of Science | patient education AND older adult AND oncology |
| PsycINFO | (older adult OR elderly OR aged OR geriatric patients) AND (oncology OR neoplasm OR newly diagnosed cancer) AND (patient education OR self care OR program development OR professional patient relation OR patient counseling OR self management OR patient centered approach OR educational needs OR patient education program OR patient education intervention OR patient psychosocial intervention) |
